# Access to care during labor and delivery and safety to maternal health*

**DOI:** 10.1590/1518-8345.3470.3292

**Published:** 2020-06-08

**Authors:** Juliana Vicente de Oliveira Franchi, Sandra Marisa Pelloso, Rosângela Aparecida Pimenta Ferrari, Alexandrina Aparecida Maciel Cardelli

**Affiliations:** 1 Universidade Estadual de Londrina, Centro de Ciências da Saúde, Londrina, PR, Brazil.; 2 Universidade Estadual de Maringá, Departamento de Enfermagem, Maringá, PR, Brazil.; 3 Universidade Estadual de Londrina, Departamento de Enfermagem, Londrina, PR, Brazil.

**Keywords:** Health Services Accessibility, Maternal-Child Health Services, Quality of Health Care, Maternal Health, Parturition, Patient Safety, Acesso aos Serviços de Saúde, Serviços de Saúde Materno-Infantil, Qualidade da Assistência à Saúde, Saúde Materna, Parto, Segurança do Paciente, Accesibilidad a los Servicios de Salud, Servicios de Salud Materno-Infantil, Calidad de la Atención de Salud, Salud Materna, Parto, Seguridad del Paciente

## Abstract

**Objective:**

to analyze the time of access to care during labor and delivery and the safety of maternal health.

**Method:**

cross-sectional analytical study, carried out in five maternity hospitals, four of which are of habitual and intermediate risk and one of high risk. For data collection, data from the maternal medical record and interviews with the puerperal woman were used. In the data analysis, the Chi-square test (p≤0.05) was performed to search for possible associations between the independent variables - model three delays and dependents [Adverse maternal outcomes], [Knowledge about labor/delivery] and [Service satisfaction].

**Results:**

statistical significance was observed between the adverse maternal outcome and the delay in looking for a health service (p = 0.005) and the delay in transport to the maternity hospital (p = 0.050), while the outcome knowledge about labor/delivery showed statistical association with delay in looking for a health service (p = 0.048). There was no statistically significant difference between the three delays model and satisfaction with the care.

**Conclusion:**

the women’s knowledge about labor and delivery and the time of access to obstetric care negatively interferes with the maternal outcome at delivery, which directly impacts maternal health safety.

## Abstract (pt)

**Objetivo:**

analisar o tempo de acesso aos cuidados no trabalho de parto e no parto e a segurança para a saúde materna.

**Método:**

estudo transversal analítico, realizado em cinco maternidades, sendo quatro maternidades de risco habitual e intermediário e uma de alto risco. Para coleta de dados utilizou-se dados do prontuário materno e entrevista com a puérpera. Na análise dos dados foi realizado o Teste Qui-quadrado (p≤0,05) para busca de possíveis associações entre as variáveis independentes - modelo três atrasos e dependentes [Desfechos maternos adversos], [Conhecimento sobre trabalho de parto/parto] e [Satisfação no atendimento].

**Resultados:**

observou-se significância estatística entre o desfecho materno adverso e o atraso em procurar por um serviço de saúde (p=0,005) e o atraso no transporte até a maternidade (p=0,050), enquanto o desfecho conhecimento sobre trabalho de parto/parto apresentou associação estatística com atraso em procurar por um serviço de saúde (p=0,048). Não houve diferença estatisticamente significativa entre o modelo três atrasos e a satisfação no atendimento.

**Conclusão:**

o conhecimento das mulheres sobre o trabalho de parto e parto e o tempo de acesso aos cuidados obstétricos interfere negativamente no desfecho materno no parto o que impacta diretamente na segurança à saúde materna.

## Abstract (es)

**Objetivo:**

analizar el tiempo de acceso a los cuidados de salud en el trabajo de parto y parto y su incidencia en la seguridad para la salud materna.

**Método:**

estudio transversal analítico, realizado en cinco maternidades de riesgo habitual e intermedio y en una de alto riesgo. Para la recolección de datos, se utilizaron los datos de la historia clínica materna y la entrevista realizada con la puérpera. En el análisis de datos se realizó la Prueba de Chi-cuadrado (p≤0,05) para la búsqueda de posibles asociaciones entre las variables independientes- modelo de tres demoras- y dependientes [Resultados maternos adversos], [Conocimiento sobre trabajo de parto/parto] y [Satisfacción en la atención]

**Resultados:**

se observó relevancia estadística entre el resultado materno adverso y la demora en buscar atención en un centro de salud y la demora en el transporte hasta la maternidad (p=0,050), mientras que la variable relativa al conocimiento sobre trabajo de parto presentó asociación estadística con la demora en buscar un centro de salud (p=0,048). No hubo diferencias significativas entre el modelo de las tres demoras y la satisfacción en la atención.

**Conclusión:**

el conocimiento de las mujeres sobre trabajo de parto y parto y el tiempo de acceso a la atención obstétrica tienen una incidencia negativa en el resultado materno durante el parto, situación que impacta de manera directa sobre la seguridad de la salud materna.

## Introduction

Safety and obstetric quality are emerging and important topics in the current health scenario, in addition to the pressure and expectations of patients and families for positive results, health service managers are also concerned with harmless care and better care outcomes being provided to the population^(^ 1 ^)^.

In this context, an important factor for the safety and quality of maternal care is the timely access to obstetric services, because the delay in receiving obstetric care is significantly associated with the severity of adverse maternal outcomes^(^ 2 ^)^.

The authors developed a model called “three delays” to assess access to obstetric care, broken down into three components or phases: (1) delay in the decision to look for the health service; (2) delay in identifying and reaching the appropriate health service; and (3) delay in receiving adequate care at the appropriate time. Since then, this model has been used to explain most fatal maternal outcomes^(^ 3 ^)^ and some cases of severe maternal morbidity^(^ 2 ^,^ 4 ^)^.

Therefore, it is worth mentioning that in addition to maternal death and life threatening situations (*near miss* maternal), there are other incidents that cause adverse maternal outcomes of lesser severity that cannot be left aside, as they are routinely present in childbirth’s reality^(^ 5 ^)^. Therefore, when there is any obstetric complication during labor and delivery, the importance of recognizing danger signs, the quick search for care and the provision of adequate care in a timely manner are fundamental to safety maternal health.

So, considering that, in Brazil: 98% of deliveries occur within hospital health organizations^(^ 6 ^)^; that timely access to obstetric services significantly reduces the number of fatal adverse maternal outcomes, with positive results for maternal health safety^(^ 7 ^)^; that maternal and child health policies to promote the promotion of service quality, such as the *Rede Mãe Paranaense* program whose mission is to guarantee access and health care, promoting safe and quality care during pregnancy, childbirth, the puerperium and children under one year old^(^ 8 ^)^, the studies that include access to maternity hospitals for diagnosing the quality of care and patient safety.

One hopes, with this study, to point out the existing gaps for achieving appropriate and quality obstetric care, providing support for managers to implement actions that strengthen public health policies that advocate maternal access to obstetric services. In addition, investments in health education are expected to be directed towards this specific population, in order to guarantee quality and safety in care, right of access to health and user information.

Considering the relevance of recognizing, and responding in a timely manner, obstetric care to improve maternal outcomes and safety in childbirth care, this study aimed to analyze the time of access to care during labor and delivery and the safety to maternal health.

## Method

This is an analytical cross-sectional study nested in the prospective cohort, carried out in five hospitals/maternities enrolled within the *Rede Mãe Paranaense* program located in the 17^th^ Regional Health Region of the state of Paraná, characterized by maternity hospitals that attend pregnant women under Habitual Risk (HBR) and Intermediate Risk (IR) (four maternities without Neonatal/Pediatric and Adult Intensive Care Units in the municipality of Rolândia, Ibiporã, Cambé and Londrina) and reference maternity hospitals for High Risk (HR) pregnancies (one maternity unit with Neonatal/Pediatric Intensive and adult Care Unit in the city of Londrina).

The study population consisted of postpartum women admitted to these maternity hospitals and based on stratified sampling, each institution is based on the number of births that took place in the maternity hospitals in 2016. The minimum sample size of 386 mothers was estimated, to which 20%was added, as a safety margin to meet the sample number and considering possible losses, obtaining an N=470, which were distributed between the HBR and IR maternity hospital of Rolândia IR (n=59), Ibiporã (n=63), Cambé (n=49), Londrina (n=230) and the Londrina HR maternity hospital (n=69).

The inclusion criterion was women with a gestational age of ≥ 32 weeks and living in the urban area of the municipalities of the 17^th^ Regional Health Region of Londrina.

A pilot study was carried out to adapt the instrument, train and monitor undergraduate nursing students in order to instruct them in the data collection steps. The amount of the pilot test was not included in the final sample.

Sequentially, data collection started from July 2017 to March 2018. A structured instrument was used to search for information available in the maternal medical record and interview with the mothers. Information was collected on previous socioeconomic, demographic, obstetric data, and access to health services according to the model of three delays^(^ 3 ^)^ in order to analyze the time of access to obstetric care and the occurrence of adverse maternal outcome; knowledge about labor and delivery and user satisfaction in care, obtained through obstetric care indicators, knowledge about labor and delivery and satisfaction in care.

The outcome of knowledge on labor and delivery was considered positive when women answered affirmatively about guidelines related to labor and delivery, clarification of all desired information and absence of doubts about the evolution and care in labor and delivery. Satisfaction outcome with care during labor and delivery was verified by means of a dichotomous variable (satisfied and dissatisfied) followed by a question about the reason for their (un) satisfaction.

The three delays model refers to women’s access to health services: 1^st^ - delay in deciding to seek health care; 2^nd^ - delay in transportation to maternity; and 3^rd^ - delay in the attendance of the health professional^(^ 3 ^)^. In this study, the unit of analysis was not the delay, but the “access time” to health care, based on the reports of the mothers, based on the three phases proposed by the authors. The term access was used for the act of receiving assistance in the childbirth care by obstetric services in a timely manner, because these weaknesses impair care and can contribute to the occurrence of adverse maternal outcomes, as previously described. It is noteworthy that the beginning of the measurement of time was considered the beginning of the symptoms of labor.

To minimize the data collection bias and guarantee the quality of the information, a collection script was created as a standard operating procedure adopted by all collectors. Double data collection was also performed, one for data from medical records and another for interviews with the puerperal woman. Subsequently, data entry was performed by people trained in how to handle the program, and performed by pairs, using double entry for greater reliability of the results.

Data were compiled in the Statistical Package for Social Sciences (SPSS), version 22.0, and analyzed using the Chi-square test, to search for possible associations (p <0.05) between independent variables (three delays model) and dependent ones. The dependent variables were: adverse maternal outcome, knowledge about labor and delivery and satisfaction in delivery care.

This study respected the ethical precepts of the individuals involved, according to Resolution No. 466/12 of the National Health Council^(^ 9 ^)^.

This study is an excerpt from the metacentric research project funded by the National Council for Scientific and Technological Development (CNPq).

## Results

As for the socioeconomic and demographic characteristics that the majority of puerperal women were aged between 20 and 34 years old (70.2%), more than 12 years of study (46.0%), unpaid occupation (58.5%), family income two to three minimum wages (54.7%) and had a partner (86.6%). Regarding the previous clinical-obstetric history, it was found that 67.9%of women had no pathological clinical-obstetric history, and 52.8% were primiparous.

Table 1 shows the guidelines received and behaviors taken by women regarding access to the place of delivery and the type of transportation used to the maternity ward. Among the guidelines, 84.9% of women were informed which hospital to look for in an emergency, 78.7% did not visit the maternity hospital before delivery, 79.4% had their home address, and 75.5% used the car suitable for transportation to maternity.

**Table 1:** Distribution of the guidelines received and conducts taken by women regarding access to the place of delivery and the type of transportation used to the maternity hospital. Paraná, Brazil, 2018

	N	%
It was informed which hospital to look for in an emergency		
Yes	399	84.9
No	71	15.1
Visited the maternity ward before delivery		
Yes	100	21.3
No	370	78.7
Where did you come from to the maternity hospital?		
UBS*	37	7.8
City hospital	44	9.5
Residence	373	79.4
Other	16	3.3
Type of transport used to come to the maternity hospital		
SAMU^†^	73	15.5
Simple ambulance	25	5.3
Own car	355	75.5
Other	17	3.7
Total	470	100.0

* UBS = Basic Health Unit;

† SAMU = Mobile emergency care service

In the period of data collection, an adverse maternal outcome (AMO) developed in 46.8% (n = 220) of the purperous, distributed in the five maternity hospitals in this study.

Table 2 shows the types of adverse maternal outcomes developed during hospitalization for the childbirth, defined as events with damages that interfere in the *continuum* of the maternal health. Among the AMOs, the intrapartum complication represented the majority (38.2%) of the cases, followed by the 3^rd^ and 4^th^ degree lacerations (27.3%) (Table 2).

**Table 2:** Types of adverse maternal outcomes developed during hospitalization for delivery. Paraná, Brazil, 2018 n=220

	N	%
Disease developed in the non-hemorrhagic pre-delivery	10	4.5
Disease developed in the infectious pre-delivery	24	10.9
Disease developed in hypertensive pre-delivery	28	12.7
Disease developed in the pre-delivery clinical condition	02	1.0
Disease developed in the metabolic pre-delivery	02	1.0
3rd and 4th degree perineum laceration	60	27.3
Intrapartum complications *	83	38.2
Complications fourth period (Greenberg)^†^	11	4.5
Total	220	100.0

* n =83 (56 progression dystocia, 02 eclampsia, 03 placental abruption, 18 fetal distress, 04 others);

† n = 10 (02 eclampsia, 06 postpartum hemorrhage, 02 uterine massage, 01 removal of retained products)

In this study, the outcome of women’s knowledge about labor and delivery was also investigated, and it was found that 57.2% (n = 269) of the puerperal women were aware of the care and guidelines of that period. The outcome of satisfaction in care was also analyzed, and it was identified that 89.5% (n=421) of the puerperal women were satisfied with the care received during hospitalization for childbirth.

Table 3 shows the distribution of access time to obstetric care according to the three delays model, and the adverse maternal outcome. There was a statistically significant difference between the first delay - time to decide to seek a health service (p = 0.005); second delay - transport time to maternity (p=0.050) and the AMO, with 63% of women who referred more than nine hours after the onset of symptoms to seek obstetric service developed an adverse maternal outcome; and 61.7% of women who reported more than 30 minutes in the transport to the maternity hospital had AMO during hospitalization for delivery.

**Table 3:** Distribution of access time according to the 3 delays model by Thaddeus and Maine (1994) and the adverse maternal outcome. Paraná, Brazil, 2018

Variables	Adverse maternal outcome				
	Yes		No		
	N	%	N	%	p-value
Decision to seek care (1st delay) ^†^ ^‡^					
Up to 3 hours	109	48.9	114	51.1	p = 0.005
4 to 8 hours	22	34.9	41	65.1	
Above 9 hours	46	63.0	27	37.0	
Transport time to the health service (2nd delay) ^†^					
Up to 30 minutes	146	49.7	148	50.3	p = 0.050
31 min to 1h 30 min	50	61.7	31	38.3	
Waiting time for medical care (3rd delay)^†^					
Up to 15 minutes	111	48.7	117	51.3	p = 0.362
16 to 30 minutes	36	52.2	33	47.8	
31 min to 1 hour	32	41.0	46	59.0	

* Qui-square test (p<0.05);

† n = 375 (95 women were excluded being hospitalized for realization of elective/iterative caesarean section);

‡ No record – 16 women

Regarding the distribution of access time to obstetric care, according to the model three delays and knowledge about labor and delivery, there was statistical significance between the first delay - time to decide to seek care (p=0.048) and the outcome knowledge on labor and delivery. Knowledge about labor and delivery was found in 60.1% of women who took less than three hours from the onset of symptoms to seek health care (Table 4).

**Table 4:** Distribution of access time according to the 3 delays model by Thaddeus and Maine (1994) and the outcome of knowledge on labor and delivery. Paraná, Brazil, 2018

Variables	Knowledge on labor and delivery				
	Yes		No		
	N	%	N	%	p-value
Decision to seek care (1st delay)^†^ ^‡^					
Up to 3 hours	134	60.1	89	39.9	p = 0.048
4 to 8 hours	45	71.4	18	28.6	
Above 9 hours	37	50.7	36	49.7	
Transport time to the health service (2nd delay)†					
Up to 30 minutes	178	60.2	117	39.8	p = 0.523
31 min to 1 h 30 min	45	56.3	35	43.8	
Waiting time for medical care (3rd delay)†					
Up to 15 minutes	145	63.2	84	36.8	p = 0.143
16 to 30 minutes	35	50.7	34	49.3	
31 min to 1 hour	43	55.8	34	44.2	

* Chi-square test (p<0.05);

† n = 375 (95 women who were admitted for elective/iterative cesarean section were excluded);

‡ No record – 16 women

There was no statistical difference between the time of access to obstetric care, according to the 3 delays model, and the outcome satisfaction in care.

## Discussion

The three delays model was created to explain cases of obstetric emergencies that contribute to the occurrence of fatal maternal outcomes, but other situations of less serious maternal outcomes can also evolve unfavorably, leading to an urgency and \/or obstetric emergency that represent risks to maternal health therefore, they should also be investigated, as they are more prevalent in the current obstetric care scenario^(^ 5 ^)^.

The factors that influence this model in relation to the time of access to obstetric care and the maternal outcome in the 1st delay are the socioeconomic and cultural conditions of women, accessibility to health services and the quality of care received previously; in the 2^nd^ delay, accessibility to health services; and, finally, on the 3^rd^ delay, the quality of care offered^(^ 3 ^)^.

A survey that evaluated 3 delays model in a Brazilian public hospital found that most women experienced the first delay after the onset of labor pains. According to these women, the husband’s late decision to seek care and health professionals was responsible for the delay. For the second delay, he found that the delay in access was due to distance and traffic. The third delay was experienced by the high demand of patients in an obstetric emergency. Furthermore, the lack of knowledge and precarious socioeconomic factors were also identified as causing these delays^(^ 10 ^)^.

In this study, women who took more time to make the decision to seek health care and more transport time to maternity had a negative association regarding the occurrence of adverse maternal outcome, which is, they developed a greater number of AMOs.

These findings are in line with an international study, in which women who arrived at the hospital within four hours of the decision to seek care had a greater chance of a favorable outcome than those who arrived within eight hours^(^ 11 ^)^.

It is known that women’s decisions to seek assistance during labor and birth are influenced by their lack of preparation for birth, family context, personal beliefs and perceptions, limitations of intra-structure, geographical location and inappropriate attitudes of professionals, which favor delay in reaching obstetric care^(^ 12 ^)^.

In this sense, another research developed outside Brazil found that there is a disarticulation between the health team and women regarding the first delay, represented by the inadequate knowledge of these women. And, that the structural barriers for accessing the services may limit your options for seeking cares. These weaknesses can result in neglect of more systemic failures that are the central cause of the problem, serving as a threat to maternal health safety^(^ 13 ^)^.

Specifically in relation to the first delay, when obstetric emergencies arise during labor and delivery, the importance of recognizing danger signs and seeking care quickly is essential for responding in a timely manner and improving maternal and neonatal outcomes^(^ 7 ^)^. Furthermore, knowledge about the current clinical condition and the woman’s guidance on the need for care are essential for the decision to seek a health service or call for care in a timely manner^(^ 14 ^)^.

Another data found in this study was the longer time spent transporting to the maternity hospital and the occurrence of AMOs. Similar findings were found in a study in southeastern Brazil, which analyzed the fatal maternal outcome and accessibility to health services, identifying that the distance between home and hospital is an important risk factor for the fatal outcome in the studied population^(^ 15 ^)^.

A study carried out in another region of the country also highlighted that inadequate access to care in childbirth care can increase maternal risks, due to possible obstetric complications that can be avoided by timely care, leading to severe complications when unavailable^(^ 16 ^)^.

A situation that deserves to be highlighted in the access to obstetric care is the referral, in a timely manner, to services that offer adequate health care, according to the needs of women, which contributes positively to quality and safe care for women.

Thus, even in the face of a low risk of adverse events occurring in the care provided, an ongoing awareness of the possible presentation of high risk complications should be part of the daily practice of obstetric services^(^ 14 ^)^.

This study also identified that although a significant portion of women received guidance on which maternity hospital to seek and the availability of mobile service in an emergency, less than a third of them were transported to the obstetric service by ambulance, as two thirds had access to the maternity hospital on their own, which may have contributed to the increase in the decision time to look for the health service and the transportation time to the maternity hospital.

An international systematic review of the qualitative evidence of facilitators and barriers of care in birthplaces in low and middle income countries, identified that the key barriers are influenced by the perception of labor and childbirth, by socio-cultural factors, by the quality of care in the childbirth and availability and access to resources, the latter in relation to long distance to maternity, lack of accessible transport, delay in accessing a reference service for childbirth and indirect costs related to birth places^(^ 17 ^)^.

Similar to these data, a study showed that obstacles to obstetric care were represented by the lack of adequate delivery places, insufficient transport and inadequate supply of health care providers, which resulted in adverse maternal outcomes in developing countries^(^ 7 ^)^.

The difficulties in the demand and in the supply that hinder the use of health services referring to childbirth are considered primary barriers, characterized by women’s fear of being neglected or mistreated by professionals, long distance, poverty, lack of support from their husband, staff-related health system deficiency, inadequate training and inefficient referral systems^(^ 18 ^)^.

Another study carried out in an underdeveloped country pointed out that the long waiting time for care, poor quality of care, lack of privacy in obstetric services and the difficulty in the availability of adequate transport were considered the main barriers in the health system for equitable access and use of services by the population studied^(^ 19 ^)^.

In Brazil, a multicentric survey, involving 27 referral obstetric units for all regions of the country, observed that the majority of delays in receiving obstetric care was related to the accessibility of health services that showed a significant association with the increased severity of the maternal outcome^(^ 2 ^)^. And, when one evaluated the right of universality in the access of women during pregnancy and the puerperium it was found that the federal policy on maternal and child health has weaknesses in access to care for women in prenatal care, childbirth and the puerperium, therefore a study recommended that managers prioritize actions to improve this policy to guarantee access to health of the binomial^(^ 20 ^)^.

Although most of the aforementioned studies point to the availability and access to maternity hospitals as the main contributing factors to the increase in the time to receive obstetric care, it is surprising how much the woman’s experience in the care received previously influences her decision to seek the obstetric health service. Investments are essential to improve the quality of care, as one of the strategies for reducing barriers to access to childbirth care and improving women’s satisfaction with the care.

Another unprecedented finding in this study was the investigation of the time of access to obstetric care according to the three delays model and the woman’s knowledge of the evolution and care in labor and delivery. It was found that the majority of the women who reported more time to make a decision to look for a health service and a longer waiting time for care at the health service were not aware of the care during the period of labor and delivery.

Similar results were found in researches that showed that women who received information on pregnancy and childbirth were significantly more likely to know any danger signs, compared to women who did not receive information^(^ 21 ^)^; and identified a low level of women’s knowledge about obstetric danger signs during pregnancy, childbirth and postpartum, finding that women who were unaware were more susceptible to delay in deciding to seek care. This can be explained due to the failure in communication between the healthcare team and the patient^(^ 22 ^)^.

Patient-centered communication facilitates the identification of potential error and correction of health conditions during labor and delivery by professionals, patients and family members. This effective communication between professionals and patients provides safe and highly reliable care for women^(^ 23 ^)^.

Knowledge about their health condition and the care appropriate to their needs increases the autonomy of women in making decisions about their own health, developing greater confidence and awareness to seek health services that offer the best care^(^ 24 ^)^.

Delay in recognizing clinical conditions is considered one of the causes that leads to delay in seeking care. However, the failure to recognize can also occur in health services where professionals are unable to provide guidance and adequate referral. That is why the importance of health education for communities and families, in order to help them recognize the danger signs in pregnancy and the risk conditions for women and babies^(^ 25 ^)^.

For safety in childbirth care, it is necessary to establish shared responsibility, in which professionals and women are held responsible for the care process, creating environments where everyone is free to discuss and expose their safety concerns and all aspects of care, and also be encouraged to do so^(^ 26 ^)^.

The absence of a negative association between the time of access to obstetric care, according to the three delays model and the outcome of satisfaction with care, was a positive finding that can be explained by the fact that there was no significant delay in obstetric care, which commonly generates greater dissatisfaction in the health service user.

The limitation of this study is its transversal character, which reflects a specific local reality of the women studied, thus, the results cannot be generalized for the entire Brazilian population. However, it is justified because it is a significant sample of this population, the results being representative of all women who had deliveries in the maternity hospitals under study, and can also be replicated in other places of interest.

However, the reduction in adverse maternal outcomes goes beyond improving the quality of obstetric care, with no less important being the reduction in the interval between the onset of a complication and appropriate care^(^ 25 ^)^.

Weaknesses in relation to this access and the assistance provided must be identified in order to define intervention strategies in order to reduce social inequalities and contribute to the improvement of maternal outcomes^(^ 27 ^)^. Thus, the results of the present study should be seen from the perspective of assessing the quality of maternal care during delivery.

This research contributes to the advancement of scientific knowledge because it points out the existing gaps for the achievement of appropriate and timely obstetric care. In addition, the results of this study can serve as a foundation for redirecting public health policies that advocate maternal access to obstetric services, information to the user and patient safety in the obstetric area.

## Conclusion

Currently, there are studies that explain the relationship between the model three delays with maternal death and a significant part of the cases of maternal *near miss* ^.^ However, this is the first study that relates the time of access to obstetric care based on the elements of the model three delays and the occurrence of minor maternal adverse outcomes, which are also harmful to maternal health and must receive adequate and timely care in order to avoid progression to a more serious or even fatal maternal outcome.

In this study, it was observed that the time of access to obstetric care negatively interferes with the maternal outcome at delivery, even in situations without imminent risk of death. Another important finding was that the decision time to seek health care is associated with the degree of maternal knowledge, which is of great importance in providing guidance to women throughout the gestational period, during labor and delivery.

Thus, it is emphasized that health education becomes essential throughout the pregnancy-puerperal cycle, in order to contribute to decision making, access to care in a timely manner, greater information and maternal autonomy in the process of care, providing safety and a positive service experience.

In addition to access to information, it is necessary to invest in managers, in aspects related to the user’s access to obstetric services, through articulations between the care networks, in order to minimize the risks of adverse maternal outcomes that may arise due to failure in planning and organizing maternal care.

In addition, the adoption of effective practices for managing childbirth, according to current recommendations, considering care centered on women, are essential to provide quality care and a positive experience with childbirth, contributing positively to decision-making and access to obstetric care.

It is concluded that the time of access to obstetric care is significantly associated with the adverse maternal outcome and the maternal knowledge about labor and delivery, which directly impacts the quality of care and the safety of maternal health.
